# Elucidation of the anti-lung cancer mechanism of Juan-Liu-San-Jie prescription based on network pharmacology and experimental validation

**DOI:** 10.1016/j.heliyon.2023.e18298

**Published:** 2023-07-23

**Authors:** Yuli Wang, Yanbin Pan, Yingbin Luo, Jianchun Wu, Zhihong Fang, Wenjing Teng, Yu Guan, Yan Li

**Affiliations:** a Clinical Medical Center of Oncology, Shanghai Municipal Hospital of Traditional Chinese Medicine, Shanghai University of Traditional Chinese Medicine, Shanghai, China; b Diagnostic Laboratory for Hematological Diseases, Shanghai Municipal Hospital of Traditional Chinese Medicine, Shanghai University of Traditional Chinese Medicine, Shanghai, China

**Keywords:** Juan-Liu-San-Jie prescription, Traditional Chinese medicine, Lung adenocarcinoma, Apoptosis, PI3K/Akt signaling pathway

## Abstract

Lung cancer is a malignancy characterized by high morbidity and mortality, with lung adenocarcinoma being the most prevalent subtype. Our preliminary studies have demonstrated that the Juan-Liu-San-Jie (JLSJ) prescription, a Traditional Chinese Medicine prescription, possesses anti-lung adenocarcinoma cancer properties. However, the molecular mechanism underlying the therapeutic effects of the JLSJ prescription for lung adenocarcinoma remains incompletely elucidated. To address the knowledge gap, the present study employed network pharmacology to identify potential therapeutic targets. Specifically, the study utilized TCMSP, TCMID, and related references, as well as ChemMapper, to identify and predict the main active components and potential targets. Additionally, differentially expressed genes associated with the disease were obtained from the microarray dataset GSE19804 and GSE118370. The protein-protein Interaction network and Target-pathway network were then constructed. We also conducted Gene Ontology (GO) and Kyoto Encyclopedia of Genes and Genomes (KEGG) enrichment analyses, and subsequently presented the top 20 enriched pathways. The results indicated that the anti-lung cancer effects of JLSJ prescription may be attributed to its ability to mediate apoptosis of tumor cells, potentially through the PI3K/Akt signaling pathway. Then, a series of in vitro and in vivo experiments were conducted to validate the molecular mechanism predicted by network pharmacology. The findings of the in vivo study suggested that the JLSJ prescription could inhibit the growth of xenograft tumors of lung adenocarcinoma with fewer adverse effects. Also, the in vitro experiments corroborated that the JLSJ prescription could induce apoptosis of A549 cells. Furthermore, the upregulation of pro-apoptosis-related proteins and mRNAs, coupled with the downregulation of anti-apoptotic-related proteins and mRNAs, was observed. In conclusion, inducing apoptosis by inhibiting the PI3K/Akt signaling pathway was one of the underlying mechanisms by which the JLSJ prescription exerted its anti-lung adenocarcinoma effect.

## 1 Introduction

According to recent statistics from the United States, lung cancer is predicted to maintain its status as the leading cause of mortality for both genders [1]. Similarly, in China, lung cancer is the primary cause of cancer-related deaths in both men and women [2]. As the predominant pathologic subtype of lung cancer, non-small cell lung cancer (NSCLC) is experiencing rapid advancements in treatment stragteies, including targeted therapy and immunotherapy [3]. Despite improvements in the 5-year relative survival rate of NSCLC over time, the long-term prognosis remains unsatisfactory [4]. When diagnosed, the majority of lung cancer cases are either locally advanced or metastatic [5], necessitating the exploration of potential effective therapeutic approaches to enhance the clinical treatment of NSCLC.

Traditional Chinese Medicine (TCM) is gaining popularity in tumor treatment due to its multi-components, multi-link, multi-target, and multisystem regulation advantages [6]. In China, TCM is commonly combined with modern medicine to improve efficacy and reduce adverse reactions in oncology clinical practice. The JLSJ prescription, developed by Professor Li Yan, the director of the oncology department of Shanghai Municipal Hospital of Traditional Chinese Medicine, was formulated based on the TCM theory of “dispelling pathogenic factors”. The JLSJ prescription comprises two distinct herb groups that exhibit varying efficacies, namely, Chinese Sage Herb, Greater Selaginella, and Bittersweet Herb, which have the effect of clearing away heat and toxins in TCM theory; Seaweed, Spica Prunellae and Concha Ostreae, which have the effect of resolving hard lamp in TCM theory. Our prior clinical observations have demonstrated that the JLSJ prescription can improve the quality of life for patients with early-stage lung adenocarcinoma who undergo surgical resection. Additionally, it can reduce the levels of negative immune cells, including myeloid-derived suppressor cells (MDSCs) and regulatory T cells (Tregs), and significantly improve the 3-year progression-free survival rate [7]. Therefore, it is necessary to investigate the potential mechanism underlying the anti-lung cancer effect of the JLSJ prescription.

In recent times, network pharmacology has emerged as a promising approach to studying the mechanism of Chinese medicine in TCM research. By leveraging multiple authoritative databases to map the network of drug-target disease, network pharmacology facilitates the exploration of the intricate and multi-faceted interactions between drugs and the human body [8]. An increasing number of researchers have employed network pharmacology to elucidate the potential antitumor mechanisms of numerous drugs that are commonly utilized in clinical practice. Examples include Cang Niu Fang Ji Decoction for hepatic cancer [9], Dan Zhi Xiao Yao Powder for breast cancer [10], Compound Kushen injection for lung cancer [11] and hepatic cancer [12], Shenqi Fuzheng injection combined with docetaxel for lung cancer [13]. Furthermore, some researches have also uncovered the underlying mechanisms of TCM therapy in various diseases, such as cardiovascular and cerebrovascular diseases [14,15], kidney diseases [16,17], and so on.

The current investigation initially examined the antitumor effect of the JLSJ prescription through in vivo experiments. Subsequently, network pharmacology was employed to forecast the potential targets and pathways of the JLSJ prescription implicated in the biological process of anti-lung cancer. Finally, a sequence of in vivo and in vitro experiments were conducted to authenticate the anticipated mechanisms. Please refer to Fig. 1 for the flowchart.

**Fig. 1:**
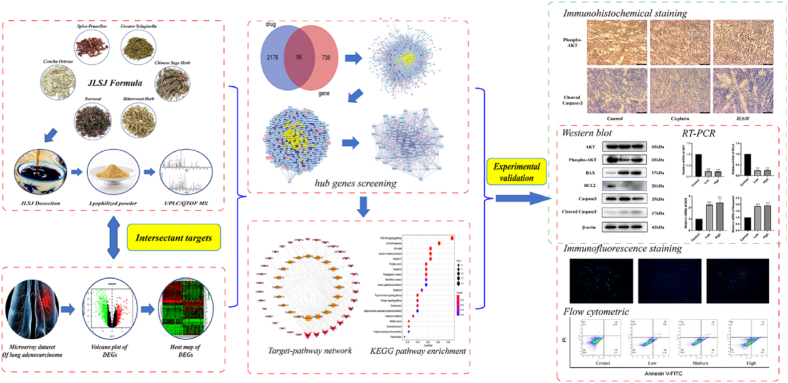
The flowchart of the present study.

## 2 Material and methods

### 2.1 Drugs

The JLSJ prescription consists of six commonly used herbs, which were shown in Table 1. For in vivo experiments, granules of six herbs were procured from the Chinese herbal pharmacy of Shanghai Municipal Hospital of TCM, which was supplied by Jiangyin Tianjiang Pharmaceutical Co, LTD. The granules were fully dissolved at 90–100 °C and the drug concentration was diluted to 2.3 g/mL with normal saline.

**Table 1:** The specific herb composition of the JLSJ prescription.

Herb	Latin scientific name	Officinal part	Dosage (g)
Chinese Sage Herb (Shi-Jian-Chuan)	*Salvia Chinensis* Herba.	Herba	30
Greater Selaginella (Shi-Shang-Bai)	*Selaginella doederleinii* Hieron.	Herba	30
Seaweed (Hai-Zao)	*Sargassum Pallidum (Turn.)* C·Ag*.*	Frond	30
Spica Prunellae (Xia-Ku-Cao)	*Prunella vulgaris* L*.*	Fruit Cluster	30
Bittersweet Herb (Bai-Ying)	*Solanum lyratum* Thunb*.*	Herba	30
Concha Ostreae (Mu-Li)	*Ostrea riuularis* Gould*.*	Shell	30

In vitro studies employed the lyophilized powder of the JLSL prescription, with the raw herbs procured from the Chinese herbal pharmacy of Shanghai Municipal Hospital of TCM and verified for quality and identification by Professor Haiqing Zhu. The specific drug dose ratio was 30 g:30 g:30 g:30 g:30 g:30 g. The JLSJ prescription extract was diluted 10-fold in deionized water and subjected to 3 h of continuous stirring at 100 °C, repeated twice, and subsequently centrifuged at 1500 g. The supernatant was collected and evaporated at 70 °C until a semisolid state was achieved. To regulate the pH value of the lyophilized powder within the range of 6–8, triethanolamine was employed as a neutralizer. The mixture was then diluted to a concentration of 1 g/mL using DMEM and stored at a temperature of −20 °C until required. The composition and quality of the lyophilized powder of the JLSJ prescription were assessed through UPLC/QTOF MS analysis. A sample of the JLSJ prescription was retained in our laboratory for future reference.

### 2.2 UPLC/QTOF MS analysis of the JLSJ prescription

UPLC/QTOF MS analysis was performed on Waters ACQUITY I-Class UPLC (Waters, Milford, MA, USA), which was equipped with a binary solvent manager, a sample manager, and a column manager. The mobile phase consisted of acetonitrile (B) and water containing 0.1% formic acid (A) following a gradient elution program: 0–2 min: 0%–2% (B); 2–22min: 2%–60% (B); 22–24min: 60%–90% (B); 24–29 min: 90% (B); 29–30 min: 90%–0% (B); 30–35min: 0% (B). The flow rate was set at 0.4 mL/min 2 ***μ***L of the test solution was injected for UPLC analysis. High-accuracy mass spectrometric data were recorded on a Waters Xevo G2-S QTOF mass spectrometer (Waters, Manchester, UK). Data acquisition was controlled by MassLynx V4.1 software (Waters Corporation, Milford, USA). Automatic metabolite characterization was performed using UNIFI 1.8 (Waters, Milford, USA) by the search of the TCM library. The specific results were shown in Supplementary Fig. 1 and Supplementary Table 1.

### 2.3 Cell lines and cell culture

The A549 cell line, derived from human non-small cell lung cancer, was obtained from the Stem Cell Bank of the Chinese Academy of Sciences. The cells were cultured in the DMEM medium supplemented with 80 U/L penicillin, 0.08 mg/mL streptomycin, and 10% fetal bovine serum. The cells were maintained in a cell incubator under standard conditions of 37 °C and 5% CO_2_.

### 2.4 Reagents

The DMEM medium and 10% fetal bovine serum were obtained from Gibco Life Technologies (Grand Island, NY, USA). The phosphate buffer saline (PBS) medium, Annexin V-FITC/PI apoptosis detection kit, and BCA protein assay kit were purchased from KeyGEN Biotech (Jiangsu, China). 5% bovine serum albumin (BSA), paraformaldehyde, electrophoresis solution, transfer solution, CCK-8 kit, RIPA were all provided by Beyotime Biotech (Beijing, China). The SDS-PAGE gels were bought from Dakewe Biotech (Shenzhen, China). RT-PCR primers were designed and provided by Sangon Biotech (Shanghai, China). Trizol reagent, Tween-20, and 20 × TBS buffer were obtained from Thermo Scientific (Rockford, IL). An ECL Kit was provided by Tanon Biotech (Shanghai, China).

### 2.5 Animal treatment with the JLSJ prescription

BALB/c (male, 18–22 g) mice aged 6–8 weeks were provided by Shanghai Cancer Institute. The mice were maintained in a temperature-controlled (22 ± 2 °C) and humidity-controlled (55 ± 5%) room under 12 h light/12 h dark cycles, with access to food and water ad libitum. The experimental operation and animal feeding were carried out in strict accordance with SPF standards. The logarithmic growth of A549 cells was initially digested with trypsin and quantified. Then, the cell concentration was adjusted at 1 × 10^7^/mL, and 200 μL of A549 suspension was subcutaneously inoculated under the skin of each mouse and monitored periodically. Upon reaching a diameter of 5 mm, the mice were randomly assigned into three groups: the control group (normal saline), the positive control group (cisplatin, administered intraperitoneally three times a week at a dose of 2.5 mg/kg each time), and the JLSJ group (administered orally at a dose of 23.2 g/kg daily). Each group was comprised of 6–8 nude mice, and the in vivo experiment was conducted over a period of 4 weeks. The mice were weighed twice a week, and tumor volume was measured using a vernier caliper. Subsequently, a growth curve was plotted. Upon completion of the experiments, all mice were humanely euthanized using CO_2_. Ethical review and approval was obtained from the Medical Ethics Committee of Shanghai Municipal Hospital of Traditional Chinese Medicine (approval number: dw2020005).

### 2.6 Network pharmacology analysis

#### 2.6.1 DEGs search, identification, and analysis

In the present study, DEGs were obtained from the microarray dataset GSE19804 and GSE118370, which were downloaded from the Gene Expression Omnibus (GEO) database (https://www.ncbi.nlm.nih.gov/geo/). 66 lung adenocarcinoma samples and 66 normal samples were analyzed by the microarray platform as GPL570 (*Homo sapiens* hgu133plus242). DEGs were screened by the Limma package of R software (version 3.6.2; http://www.r-project.org/). Fold-change (FC) in gene expression was calculated with the threshold criteria of the adjusted *P*-value < 0.05, and |log_2_FC| ≥1 was set for DEGs selection between the two groups. Heatmap and volcano plots were generated by the ggplot2 package (version 3.2.1; https://ggplot2.tidyverse.org/) of the R software. A color-coding system was used to represent the gene expression.

#### 2.6.2 Acquisition of chemical components and corresponding targets of the JLSJ prescription

The chemical components of the JLSJ prescription were obtained from TCMSP (http://tcmspw.com/tcmsp.php) [18], TCMID (http://www.megabionet.org/tcmia/) [19] and related references [20,21]. OB ≥ 30% and DL ≥ 0.18 were set as the criteria to screen the active components of the JLSL prescription. Through the ChemMapper platform (http://lilab.ecust.edu.cn/chemmapper/), we achieved the online predicted targets. Then, common targets of JLSJ prescription ingredients and lung adenocarcinoma were taken and submitted to the Uniprot database (https://www.uniprot.org/) [22], to standardize the names of potential therapeutic targets.

#### 2.6.3 PPI network construction

Through the BisoGenet (version 3.0.0) plugin [23] of Cytoscape software (version 3.7.2; http://www.cytoscape.org), PPI networks were constructed to predict the therapeutic mechanisms of the JLSJ prescription. By the CytoNCA (version 2.1.6) plugin [24] of Cytoscape, the criteria of Degree (DC) > 61 and Betweenness (BC) > 600 were applied to screen the hub genes. Finally, a new Sub-network including 52 hub genes was constructed.

#### 2.6.4 GO analysis and KEGG pathway enrichment

GO analysis attempted to describe genetic characteristics in terms of biological process (BP), cellular component (CC), and molecular function (MF). KEGG pathway enrichment analysis used data sources from known biological pathways to a gene or a set of genes with their respective KEGG pathways. GO and KEGG pathway enrichment analyses were carried out using the R/Bioconductor packages ClusterProfiler (v 3.14.3), org. Hs.eg.db (v 3.10.0) and enrichplot (v 1.6.1). A *P*-value of less than 0.05 was considered statistically significant. Use the ggplot2 package of R/Bioconductor to draw boxplots that represented the significance of the enriched pathway.

### 2.7 Immunohistochemical staining

The tumor tissues of mice were sectioned to a thickness of approximately 4 μm. Following a period of reheating at room temperature (25 °C), the tumor tissues were fixed in precooled paraformaldehyde at 4 °C for 15 min. After being washed with PBS, the sections were incubated with 3% H_2_O_2_ in the absence of light for 25 min to block endogenous peroxidase. The sections were then washed with PBS and subsequently blocked with 5% BSA for 30 min. Then, the sections were incubated overnight at 4 °C with primary antibodies of Bcl2 (Cell Signaling Technology, 4223, USA) and cleaved Caspase3 (Cell Signaling Technology, 9664, USA), followed by incubation with secondary antibodies for 1 h at room temperature. Subsequently, the sections were stained with DAB and Harris hematoxylin, and images were captured under a light microscope (magnification, × 200). Quantification of positive signals in the immunohistochemical analysis was performed using ImageJ software.

### 2.8 Cell viability assay

The CCK-8 assay was utilized to determine cell viability. A549 cells in the logarithmic growth phase were collected and seeded in duplicate in a 96-well plate at a density of 5000 cells per well. Subsequently, 100 μL of lyophilized JLSJ prescription powder was added to each well at varying concentrations of 2 mg/mL, 4 mg/mL, 6 mg/mL, 8 mg/mL, 10 mg/mL, 12 mg/mL, and 16 mg/mL. Following a 24-h incubation period, 10 μL of CCK-8 reagent was added to each well and incubated at 37 °C for approximately 2 h. The absorbance at 450 nm was measured using a microplate reader (Bio-Gene, Shanghai, China). Subsequently, the percentage of surviving cells at each concentration was determined, followed by the calculation of the IC50 value through regression curve plotting.

### 2.9 Detection of cell apoptosis by flow cytometric analysis

The Annexin V-FITC/PI apoptosis detection kit was employed to determine cell apoptosis. A total of 1 × 10^5^ logarithmic growth A549 cells were inoculated into each well of a 6-well plate. Following incubation with varying concentrations of the JLSJ prescription for 24 h, the cells were washed with PBS and stained with 5 μL of annexin V-FITC and 5 μL of Propidium Iodide (PI) for 15 min in the absence of light. The status of cell staining was analyzed using flow cytometry (Beckman Coulter, USA). Surviving cells exhibited negative results for both Annexin V-FITC and PI, while late apoptotic cells demonstrated positive results for both. Early apoptotic cells displayed positive results for Annexin V-FITC and negative results for PI. Conversely, dead cells exhibited positive results for PI and negative results for Annexin V-FITC.

### 2.10 Immunofluorescence staining

Logarithmic growth A549 cells were harvested and seeded onto a cell slide within a 24-well plate. Upon reaching a confluency of 95%–100%, varying concentrations of the JLSJ prescription were introduced. Following a 24-h incubation period, the cells were fixed with 4% paraformaldehyde for 15 min and washed with PBS. Subsequently, the cells were blocked with 5%BSA at 37 °C for 30 min and incubated overnight at 4 °C with primary polyclonal antibodies of Bcl2 (Cell Signaling Technology, 4223, USA), *p*-Akt (Cell Signaling Technology, 4060, USA), and cleaved Caspase3 (Cell Signaling Technology, 9664, USA). Then, a specific fluorescent secondary antibody was introduced in a light-deprived environment and allowed to incubate at room temperature for 1 h. Finally, DAPI was added and incubated for 5 min. The slices were then dried and sealed before being observed under a fluorescence microscope at a magnification of × 200. The fluorescence intensities were measured and quantified using the ImageJ software.

### 2.11 Real-time PCR analysis

The real-time PCR assay was developed to measure the gene expression levels of *P53*, *Bcl2*, *Bax*, and *Caspase3* genes in the tumor tissues and cell samples. The RNA was extracted from the samples using Trizol reagent and then converted to cDNA through reverse transcription. The PCR amplification procedure was carried out in accordance with the following steps: after 10 min of the pre-denaturation step at 95 °C, it then entered the denaturation-annealing-extension cycle. Among them, the denaturation step lasted for 10 s at 95 °C, the annealing step lasted for 20 s at 60 °C, the extension step lasted for 30 s at 72 °C, and 40 cycles were carried out in total. The primer sequences of the genes to be tested were designed and as followed: P53: F: 5′-GGGAGAAAACGTTAGGGTG-3′, R: 5′- CCAATCCAGGGAAGCGTG-3’; Bcl2: F: 5′-GGTGGGGTCATGTGTGTGG-3′, R: 5′-CGGTTCAGGTACTCAGTCATCC-3’; Bax: F: 5′-CCCGAGAGGTCTTTTTCCGAG-3′, R: 5′-CCAGCCCATGATGGTTCTGAT-3’; Caspase3: F: GTTTGTGTGCTTCTGAGCCA, R: TCAAGCTTGTCGGCATACTG; GAPDH: F: 5′-GGAGCGAGATCCCTCCAAAAT-3′, R: 5′-GGCTGTTGTCATACTTCTCATGG-3’. The expression level of the GAPDH gene was taken as the endogenous control, and the 2^-△△Ct^ value was used to qualify the relative gene expression levels.

### 2.12 Western blotting

The protein samples were fully lysed with RIPA lysis buffer, which contained 1% protease inhibitor and phosphatase inhibitor. Subsequently, 50 μg of protein samples were introduced into each well of the PAGE gel. The protein samples were concentrated and separated on the PAGE gel through electrophoresis and then transferred onto polyvinylidene fluoride (PVDF) membranes. After transfer, the PVDF membranes were blocked by 5% BSA for 2 h, then the following primary antibodies: PI3K (Cell Signaling Technology, 4249, USA), p-PI3K(Cell Signaling Technology, 17,366, USA), Akt (Cell Signaling Technology, 4691, USA), *p*-Akt (Cell Signaling Technology, 4060, USA), Bax (Bosterbio, USA), Bcl2 (Cell Signaling Technology, 4223, USA), P53 (Cell Signaling Technology, 2527, USA), Caspase3 (Cell Signaling Technology, 14,220, USA), cleaved Caspase3 (Cell Signaling Technology, 9664, USA) and β-Actin (Cell Signaling Technology, 4970, USA) were added and incubated at 4 °C in dark. Subsequently, the PVDF membranes were incubated with the appropriate secondary antibody at room temperature for 1 h. The protein bands were captured using a Gel Image system ver4.0 (Tanan, China), and subsequently quantified using ImageJ 6.0 software. The expression levels were normalized to β-Actin.

### 2.13 Statistical analysis

Statistical analysis and result representation were conducted using SPSS 25.0 for Windows (SPSS Inc, Chicago, IL, USA) and GraphPad Prism 8.0 (GraphPad Software Inc, California, USA), respectively. Continuous variables were presented as mean ± standard deviation (SD). The one-way analysis of variance (ANOVA) was employed to assess differences among the three groups, with statistical significance set at *P* < 0.05.

## 3 Results

### 3.1 The JLSJ prescription inhibited the growth of xenograft tumors of lung adenocarcinoma with fewer adverse effects in vivo

The results presented in Fig. 2A–C and Table 2 demonstrate a significant decrease in tumor weight and volume in both the cisplatin and JLSJ groups compared to the control group. Additionally, a reduction in the ratios of tumor to body weight was observed in the cisplatin and JLSJ groups, as depicted in Fig. 2D. While no statistical difference was observed between the cisplatin and JLSJ groups in relation to the aforementioned indices, the JLSJ group showed a trend toward superior tumor growth control. Moreover, the JLSJ treatment group exhibited higher body weights in contrast to the cisplatin group (Fig. 2E), suggesting that the JLSJ prescription yielded comparable antitumor efficacy with reduced adverse effects.

**Fig. 2:**
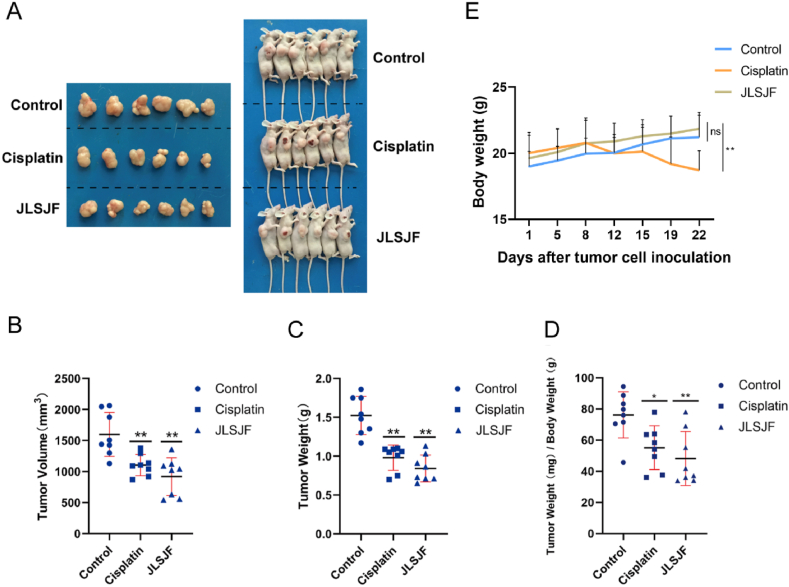
The JLSJ prescription suppressed the growth of xenograft tumors with few side effects. All values are expressed as mean ± SD, n = 6–8 for each group. (**A**) Representative images of the mice and tumors were photographed. (**B**) Tumor weights were measured. (**C**) Tumor volumes were measured. (**D**) The ratios of tumor weight to body weight were calculated. (**E**) The body weight of each group was calculated at each time point of the experiment. Time curves of body weight changes were plotted for comparasion (*P <* 0.01). ns: not significant, **P < 0.01 vs. Control.

**Table 2:** Tumor volume and weights of the subcutaneous xenograft tumor model.

	Control	Cisplatin	JLSJF
Tumor Volume (mm^3^)	1.53 ± 0.25	0.98 ± 0.16^a^	0.84 ± 0.17^a^
Tumor Weight(g)	1598.73 ± 351.42	1104.47 ± 170.91^a^	918.92 ± 303.80^a^
Tumor Weight (mg)/Body Weight(g)	76.31 ± 14.80	55.19 ± 14.07^a^	48.24 ± 17.30^a^

a *P* < 0.01 compared with the control group.

### 3.2 Analysis of differentially expressed genes (DEGs)

Firstly, a total of 132 chips were procured from the microarray dataset GSE19804 and GSE118370, which encompassed 66 lung adenocarcinoma samples and 66 normal samples. Upon conducting DEG screening analysis, a cumulative of 833 DEGs were identified between lung cancer and normal tissue. Specifically, 574 genes were observed to be down-regulated, while 259 genes were up-regulated, as depicted in Fig. 3A’s Volcano plot (Fig. 3A). The expression dataset was selected using the Limma package of R software, with the criteria of adjusted *P*-value <0.05 and |log2 (FC)|≥1. The hierarchical cluster analysis revealed the top 20 up-regulated and down-regulated genes, as shown in Fig. 3B.

**Fig. 3:**
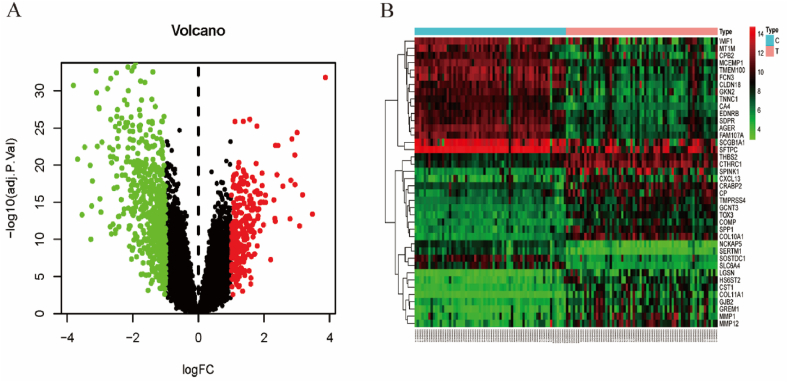
The Volcano plot and heat map of DEGs. (**A**) Volcano plot of DEGs detected in two microarray datasets. Green plots mean down-regulated DEGs; red plots mean up-regulated DEGs; black plots mean no difference. (**B**) The heat map showed the top 20 up-regulated and down-regulated genes. Red in the heat map indicated up-regulation, while green indicated down-regulation.

### 3.3 Active compounds in the JLSJ prescription

Utilizing TCMSP, TCMID, and related references, and employing screening criteria of OB ≥ 30% and DL ≥ 0.18, a comprehensive set of 112 active compounds was identified. These compounds were distributed among various herbal ingredients, with Hai-Zao, Shi-Jian-Chuan, Mu-Li, Shi-Shang-Bai, Bai-Ying, and Xia-Ku-Cao containing 17, 14, 1, 25, 9, and 46 compounds, respectively. The ChemMapper platform was utilized to predict targets for each ingredient by inputting their respective SMILE IDs.

### 3.4 Protein-protein interaction (PPI) network analysis

**Fig. 4:**
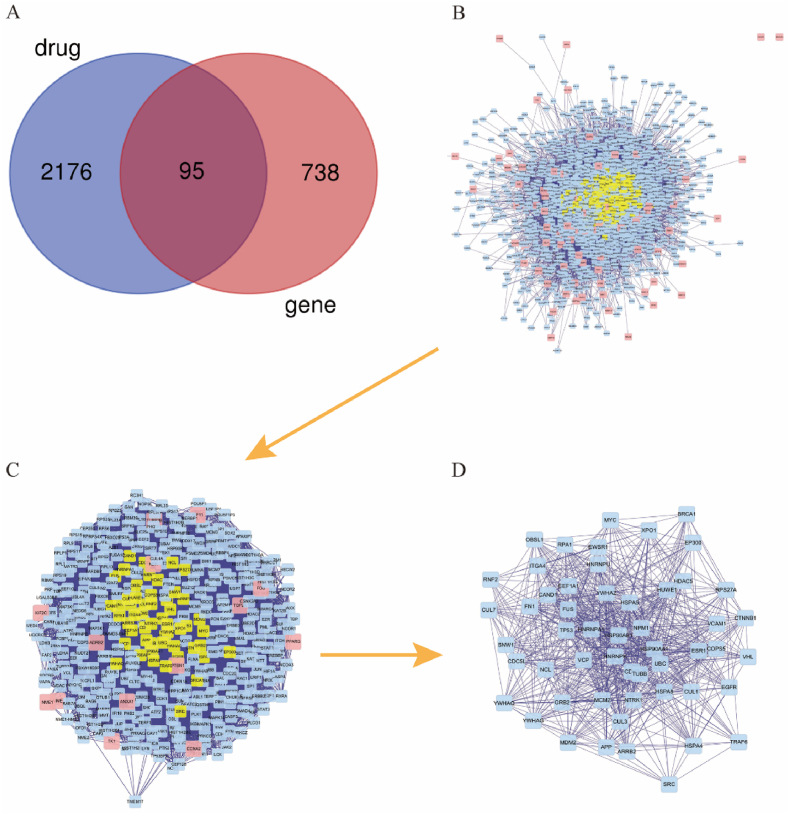
Venn diagram and PPI network. (**A**) The Venn diagram showed the intersection of disease and drug targets. (**B**) The PPI network was constructed with common target genes. (***C*-D**) Hub genes were screened out with the criteria of Degree (DC) > 61 and Betweenness (BC) > 600 successively.

Following the screening of disease target genes and their intersection with drug targets, we identified the potential therapeutic targets for each herb and the common targets shared by six herbs, as illustrated in Supplementary Figs. 2–3. The Venn diagram (Fig. 4A) demonstrated the presence of 95 common targets between DEGs of lung adenocarcinoma and potential therapeutic targets of the JLSJ prescription. Subsequently, the PPI network was constructed using the Bigenet 3.0.0 plugin of Cytoscape software 3.7.2 (Fig. 4B). The CytoNCA 2.1.6 plugin of Cytoscape was utilized to identify hub genes from the PPI network by applying the criteria of Degree (DC) > 61 and Betweenness (BC) > 600. The resulting analysis revealed a total of 52 hub genes, as depicted in Fig. 4C–D.

### 3.5 GO analysis and KEGG pathway enrichment

**Fig. 5:**
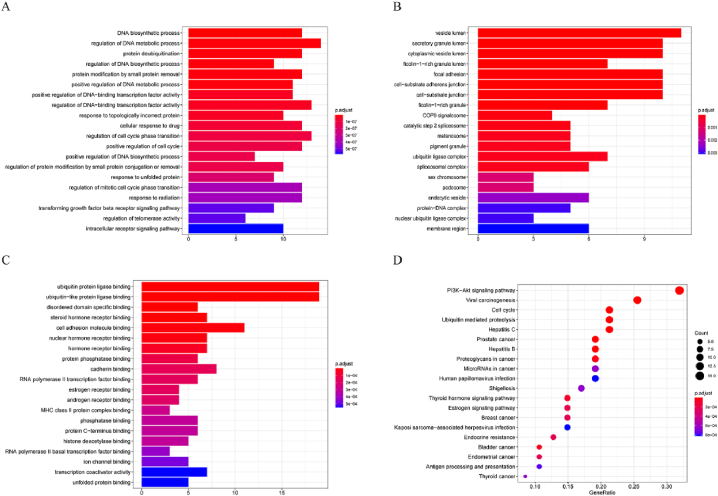
GO analysis and KEGG pathway enrichment. Bar charts showed the top 20 enriched BP **(A)**, CC **(B)**, and MF **(C)** through the GO annotation. (**D**) The Bubble chart displayed the top 20 of KEGG pathway enrichment results.

In order to investigate the functions of target genes, a GO analysis was conducted using the R/Bioconductor packages ClusterProfiler (v 3.14.3), org. Hs.eg.db (v 3.10.0) and enrichplot (v 1.6.1). The results, as depicted in Fig. 5A–C, indicate that the hub genes were significantly enriched in various categories. Specifically, for biological process (BP), the hub genes were significantly enriched in the DNA biosynthetic process and regulation of DNA metabolic process. For cellular component (CC), the hub genes were significantly enriched in vesicle lumen and secretory granule lumen. Lastly, for molecular function (MF), the hub genes were significantly enriched in ubiquitin (-like) protein ligase binding and cell adhesion molecule binding.

**Fig. 6:**
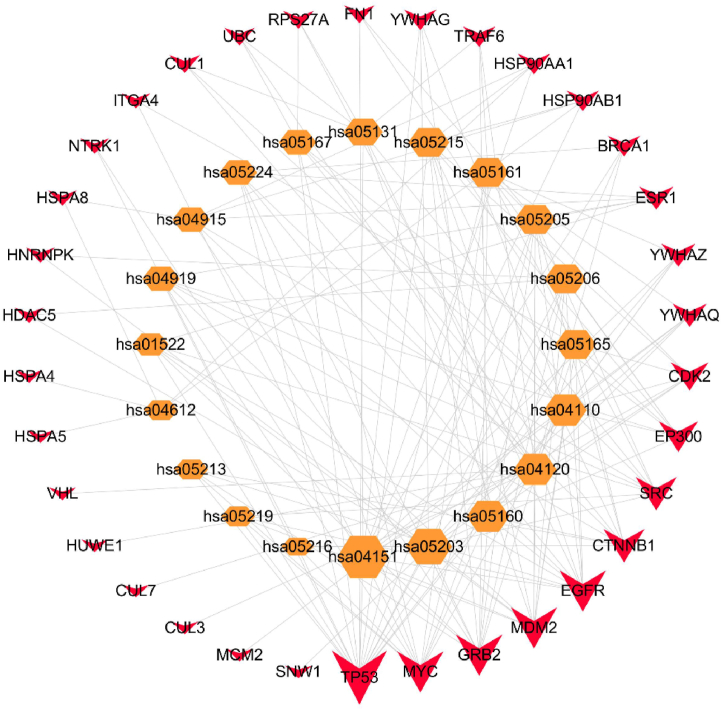
Target-pathway network. The hexagons in the inner circle represented pathway names, while the quadrilaterals in the outer circle represented the targets. The size of targets and pathways was determined by the degree of each node, which represented the biological correlation.

The present study employed R/Bioconductor packages clusterProfiler (v 3.14.3), org. Hs.eg.db (v 3.10.0) and enrichplot (v 1.6.1) to conduct KEGG pathway analysis. The findings revealed a significant enrichment of hub genes in the PI3K/Akt signaling pathway. The top 20 pathways were depicted in a bubble diagram, as presented in Fig. 5D. Furthermore, a target pathway network was established, consisting of the top 20 pathways and 34 candidate targets. Notably, TP53 emerged as the most promising anticancer target of the JLSJ prescription, closely associated with the biological process of cell apoptosis, as depicted in Fig. 6.

### 3.6 Effect of the JLSJ prescription on apoptotic related proteins involved in the PI3K/Akt signaling pathway

**Fig. 7:**
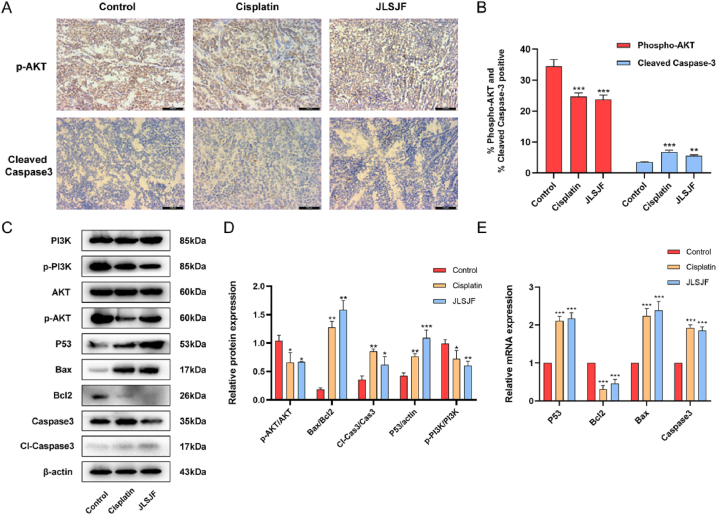
**(A**–**B)** Immunohistochemical analysis of *p*-Akt and cleaved Caspase3 expression in tumor tissues. (***C*-D**) Western blotting was performed to detect the expression levels of PI3K, p-PI3K, Akt, *p*-Akt, P53, Bax, Bcl2, Caspase3, and cleaved Caspase3 proteins in tumor tissues. (**E**) The mRNA expression of P53, Bcl2, Bax, and Caspase3 genes in tumor tissues measured by RT-PCR analysis. The values shown were the mean ± SD. *P < 0.05 vs. Control; **P < 0.01 vs. Control; ***P < 0.001 vs. Control.

To ascertain the impact of the JLSJ prescription on the apoptosis-related proteins implicated in the PI3K/Akt signaling pathway, we conducted an immunohistochemical staining analysis to determine the expression level of *p*-Akt and cleaved-Caspase3 in the tumor tissues. As illustrated in Fig. 7A–B, the expression levels of *p*-Akt were observed to decrease in both the JLSJ and cisplatin groups, while the levels of cleaved-Caspase3 protein expression were found to increase in both groups relative to the control group.

Furthermore, Western blotting analysis was conducted to evaluate the expression levels of PI3K, p-PI3K, Akt, *p*-Akt, Bcl2, Bax, Caspase3, cleaved Caspase3, and P53 in tumor tissues. The results indicated a significant increase in the ratios of cleaved-Caspase3/Caspase3, Bax/Bcl2, and P53 in both the cisplatin and JLSJ groups when compared to the control group. Conversely, the ratios of *p*-Akt/Akt and p-PI3K/PI3K were observed to decrease in both groups (Fig. 7C–D). The results of RT-PCR analysis demonstrated an upregulation in the mRNA expression levels of *P53*, *Bax*, and *Caspase3* in both the cisplatin and JLSJ groups, along with a decrease in the expression of Bcl2 in both groups, as illustrated in Fig. 7E.

### 3.7 The JLSJ prescription inhibits growth and induces apoptosis of A549 cells

**Fig. 8:**
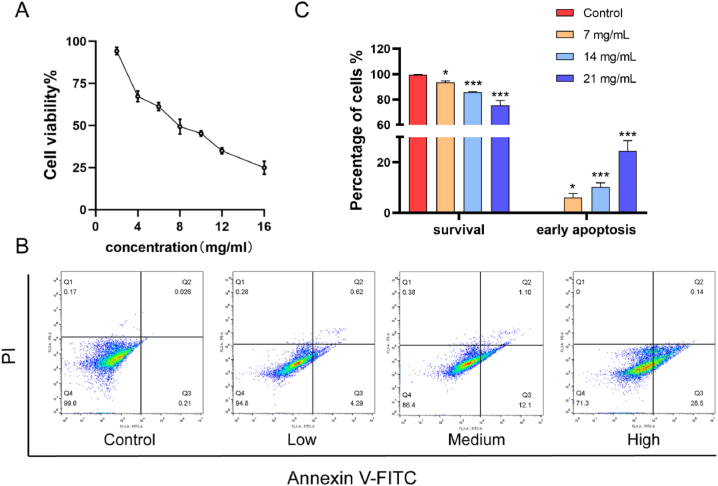
(**A**) Cell activity was detected by CCK-8 assay. (**B–C**) Cell apoptosis was detected using flow cytometry. The values shown were the mean ± SD. *P < 0.05 vs. Control; ***P < 0.001 vs. Control.

After 24-h intervention, the results indicated that cell activity was increasingly suppressed with higher drug concentration. The IC50 value was determined to be 6.961 mg/mL based on the regression curve (Fig. 8A). Subsequently, the JLSJ prescription was applied at concentrations of 7 mg/mL, 14 mg/mL, and 21 mg/mL in the flow cytometric analysis of cell apoptosis, referencing the IC50 value obtained from the CCK-8 assay. The data presented in Fig. 8B–C indicated a notable decrease in the ratio of viable cells and a significant increase in the proportion of early apoptosis cells.

### 3.8 The JLSJ prescription promotes apoptosis of A549 cells by inhibiting the PI3K/Akt signaling pathway

To provide additional evidence regarding the involvement of the JLSJ prescription in facilitating apoptosis through the PI3K/Akt signaling pathway, a sequence of in vitro experiments were executed. The JLSJ prescription's lyophilized powder was diluted to 7 mg/mL and 14 mg/mL with DMEM, serving as the low and high dose groups. Following a 24-h intervention, the cells were gathered for immunofluorescence, Western blotting, and RT-PCR assays.

**Fig. 9:**
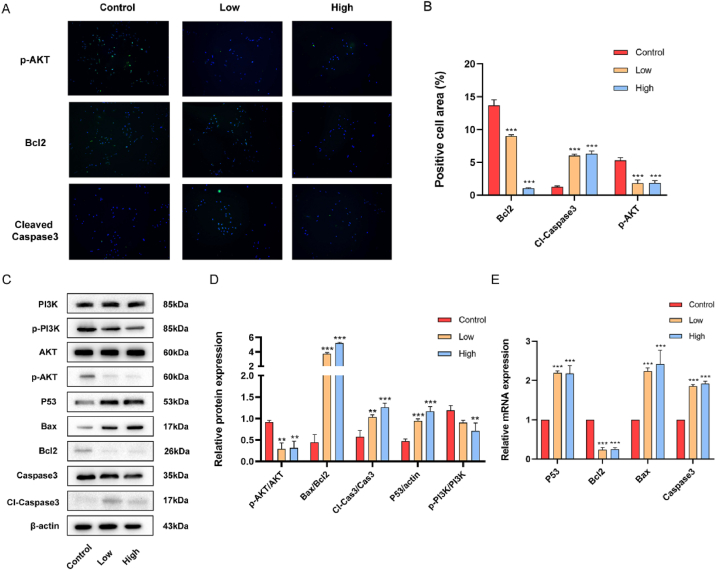
(**A-B**) Immunofluorescence staining was used to detect the expression levels of *p*-Akt, Bcl2, and cleaved Caspase3 in A549 cells. (***C*-D**) Western blotting was performed to detect the expression levels of PI3K, p-PI3K, Akt, *p*-Akt, P53, Bax, Bcl2, Caspase3, and cleaved Caspase3 proteins in A549 cells. (**E**) The mRNA expression of P53, Bcl2, Bax, and Caspase3 genes in tumor cells measured by RT-PCR analysis. The values shown were the mean ± SD. *P < 0.05 vs. Control; **P < 0.01 vs. Control; ***P < 0.001 vs. Control.

The results of the immunofluorescence assay indicated that the JLSJ prescription intervention led to an increase in the expression of cleaved Caspase3 in the cytoplasm, accompanied by a decrease in the expression of Bcl2 and *p*-Akt, as illustrated in Fig. 9A–B.

Western blotting analysis revealed that the expression levels of Bcl2, *p*-Akt, and p-PI3K decreased in both the low-dose and high-dose groups, while the expression of Bax, cleaved Caspase3, and P53 increased in both groups, as depicted in Fig. 9C–D. Additionally, RT-PCR results demonstrated a decrease in the mRNA expressions of *Bcl2* in both the low-dose and high-dose groups, whereas the expressions of *Bax*, *Caspase3*, and *P53* were observed to increase, as depicted in Fig. 9E.

## 4 Discussion

TCM has been extensively utilized in China and neighboring Asian countries for millennia to treat a diverse range of diseases. Its distinctive “holistic view” and “syndrome differentiation treatment” have made it an increasingly vital complementary therapy alongside Western medicine. Presently, as scientific research on TCM's role in the prevention and treatment of lung cancer deepens, it is increasingly employed to alleviate clinical symptoms such as fatigue, cough, expectoration [25], dyspnea, and pain [26], minimize the adverse effects of chemotherapy [27], prevent recurrence and metastasis, and extend survival [28,29].

The JLSL prescription is grounded in the theoretical framework of TCM's comprehension of the pathology of lung cancer. It is a simplified version of ‘Yi-Fei-Xiao-Ji Decoction’, a medicinal prescription employed by the Chinese medicine master *Jiaxiang Liu* for the management of lung cancer. The prescription comprises six herbs that possess a discernible antitumor effect. According to the principles of Chinese medicine, the primary pathogenic factors of lung cancer are *sputum* and *toxin*. The JLSJ prescription, which consists of *Chinese Sage Herb*, *Greater Selaginella*, *Bittersweet Herb*, is formulated to eliminate *heat* and *toxin*, while *Seaweed*, *Spica Prunellae*, *Concha Ostreae* are included to alleviate *hardness* and dissolve *sputum* with Traditional Chinese theory. Recent research has demonstrated that approach of eliminating heat and toxin can exert various anti-lung cancer effects [30].

For example, the *Greater Selaginella* contains diverse volatile oil components that exhibit noteworthy inhibitory effects on A549 cells [31]. The *Chinese Sage Herb* contains triterpenoids, namely *Ursolic Acid* or *Oleanolic acid*, which demonstrate antiproliferation, anti-invasive, and pro-apoptotic effects on human lung cancer cells [32]. Furthermore, the method of softening *hardness* and resolving *sputum* has been validated to possess the potential to inhibit tumor proliferation and metastasis, promote tumor apoptosis and improve chemotherapy resistance [33]. For instance, the *seaweed polysaccharide* found in *seaweed* has been shown to possess antitumor properties by impeding tumor cell proliferation, promoting apoptosis, and stimulating lymphocyte proliferation [34,35]. Additionally, *triterpenoids*, which are the primary antitumor active constituents of *Spica Prunellae*, exhibit direct cytotoxic effects on various lung tumor cells, including A549 and SPC-A-1 [36].

Hence, the JLSJ prescription presents a promising therapeutic approach for lung cancer. Nevertheless, the majority of prior studies have concentrated on individual herbs or monomers, and the underlying mechanism of this compound necessitates further investigation. In contrast to conventional drug research approaches, the study of TCM compounds aims to transcend the “one-drug, one-target” paradigm. Following the introduction of the “multicomponent network target” model of network pharmacology by Professor Andrew L. Hopkins, it has emerges as a convergence point in TCM research and has been progressively implemented in the study of TCM compounds [37]. Network pharmacology offers an optimal approach to comprehending the intricate impacts of herbs or compounds on the human body by utilizing the network of “drug-target-disease-pathway” interactions [38].

In this study, we first conducted in vivo experiments to investigate the tumor-suppressive effect of the JLSJ prescription. Our findings demonstrated that the JLSJ prescription significantly reduced tumor burden compared to the control group. Furthermore, the JLSJ group exhibited fewer adverse effects as indicated by the upward trend in the bodyweight of nude mice compared to the cisplatin group. To elucidate the molecular mechanisms of the JLSJ prescription, we utilized network pharmacology to predict the underlying targets and pathways. The present study applied various resources, including the TCM database, online drug target prediction tools, and GEO database, to identify potential active compounds and therapeutic targets of the JLSJ prescription. By conducting a topology analysis of the PPI network, a total of 52 hub genes were identified. Additionally, enrichment analysis was performed, resulting in the identification of 20 key pathways and 34 targets. These findings suggest that the JLSJ prescription exerts an anti-lung cancer effect through a multi-component, multi-target, and multi-pathway approach. Notably, TP53, which is involved in the PI3K/Akt signaling pathway, was identified as the most likely target of the JLSJ prescription.

The PI3K/Akt signaling pathway is integral to numerous cellular processes, including but not limited to cell proliferation, apoptosis, cell cycle regulation, cell differentiation, and angiogenesis [39], which collectively encompass the majority of tumor biological characteristics [40]. TP53, a renowned tumor suppressor, primarily governs apoptosis and the cell cycle [41]. Apoptosis, a strictly regulated form of cell death, serves as the fundamental mechanism of tumor suppression [42], while apoptosis resistance is a well-established hallmark of tumors [40]. Akt, also referred to protein kinase B, is a crucial molecule located downstream of the PI3K signaling pathway. The existence of three forms of Akt, namely Akt 1, Akt 2, and Akt 3 [43], is significant in the regulation of cell growth, proliferation, migration, and glucose metabolism [44]. Consequently, the activation of Akt is deemed indispensable for the survival and proliferation of tumor cells, as well as the acceleration of glucose metabolism and other synthetic pathways observed in cancer cells [45]. Research has demonstrated that Akt modulates the function of proteins related to apoptosis via phosphorylation. Specifically, activated Akt can phosphorylate and impede the activities of diverse apoptotic factors, including Bim, Bax, Bad, and FoxO1 [43]. Conversely, *p*-Akt can also activate the apoptotic inhibitor XIAP [46] and hinder p53-mediated apoptosis by phosphorylating MDM2 [43]. As such, the Akt pathway plays a pivotal role in regulating cell apoptosis. The inhibition of Akt phosphorylation has been demonstrated to be viable method for inducing apoptosis in tumor cells. The current investigation utilized both in vitro and in vivo experiments to reveal that the JLSJ prescription intervention resulted in a significant reduction in the expression of *p*-Akt protein relative to total Akt.

Regarding the activation patterns of apoptosis, two signaling pathways can be triggered: the endogenous pathway mediated by mitochondria and the exogenous pathway mediated by death receptors, ultimately leading to the activation of Caspase3 [47]. The Bcl2 family, which includes Bcl2 and Bcl2-related proteins, is the most notable protein family involved in regulating apoptotic cell death. Numerous members of the Bcl2 family can be categorized as either pro-apoptotic proteins, such as Bcl-Xs, Bax, Bak, Bik, Bid, and others, or anti-apoptotic proteins, such as Bcl2, Bcl-XL, Bcl-w, Mcl-1, and others [48]. Hence, alternations in the expression levels of Bcl2 and Bax are widely acknowledged as a diagnostic indicator for determining the occurrence of apoptosis in cells [49]. The findings of the present study revealed a dose-dependent increase in the overall upregulation of apoptotic cells (comprising early and late apoptosis) following the administration of the JLSJ prescription intervention. The results obtained from Western blotting and RT-PCR analyses revealed a significant decrease in the levels of anti-apoptotic protein Bcl2 and its corresponding mRNA expression, while there was a notable increase in the levels of pro-apoptotic protein Bax and its mRNA expression. Additionally, a significant increase in the levels of cleaved-Caspase3 was observed.

The objective of our experimental series was to elucidate the fundamental mechanism of the JLSJ prescription in anti-lung adenocarcinoma. Our in vitro and in vivo investigations into this mechanism have revealed that the JLSJ prescription effectively impedes Akt phosphorylation, modulates the expression of pro-apoptotic and anti-apoptotic members of the Bcl2 family, induces apoptosis in lung adenocarcinoma cells, and exerts a potent anti-tumor effect.

The present study has certain limitations that warrant mention. Firstly, due to the multitarget and multi-pathway nature of TCM compounds, it is imperative to verify other enrichment pathways in future experiments. Secondly, given the complexity of TCM compounds, it is necessary to identify the effective monomers responsible for the anti-lung cancer effect in the JLSJ prescription and investigate their functions and targets. Finally, it is crucial to conduct a scientific assessment of the acute and chronic toxicity of the JLSJ prescription, which remains to be investigated.

## 5 Conclusion

In conclusion, the inhibitory effect of the JLSJ prescription on lung adenocarcinoma has been confirmed through in vitro and in vivo data. The results, which were supported by network pharmacology prediction and experimental verification, indicate that the JLSJ prescription can induce cell apoptosis by inhibiting the PI3K/Akt signaling pathway, thereby exerting its anti-lung adenocarcinoma effect.

## Author contribution statement

Yan Li, Yu Guan: Conceived and designed the experiments.

Yu Guan: Performed the experiments.

Yuli Wang: Performed the experiments; Wrote the paper.

Yanbin Pan: Performed the experiments.

Yingbin Luo, Jianchun Wu: Analyzed and interpreted the data.

Zhihong Fang, Wenjing Teng: Contributed reagents, materials, analysis tools or data.

## Data availability statement

Data included in article/supplementary material/referenced in article.

## Ethics statement

The animal study was reviewed and approved by Animal Ethics Committee of Shanghai Municipal Hospital of Traditional Chinese Medicine (Shanghai, China).

## Declaration of competing interest

The authors declare that they have no known competing financial interests or personal relationships that could have appeared to influence the work reported in this paper.

## Figures and Tables

**figs1:**
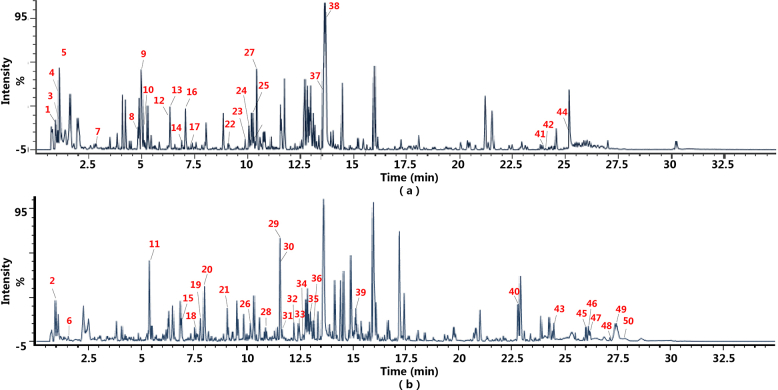


**figs2:**
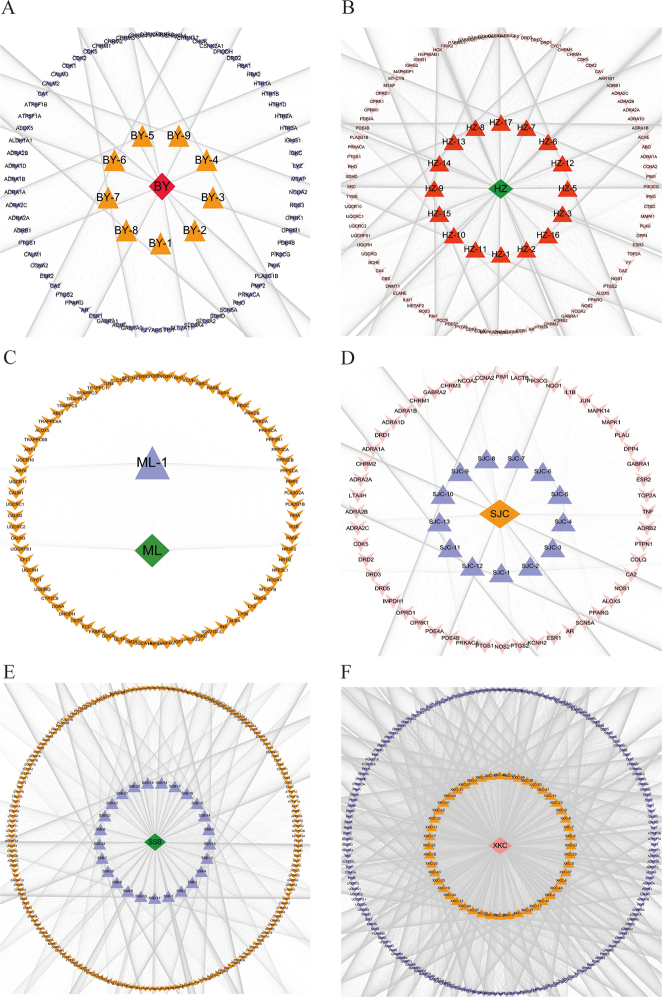


**figs3:**
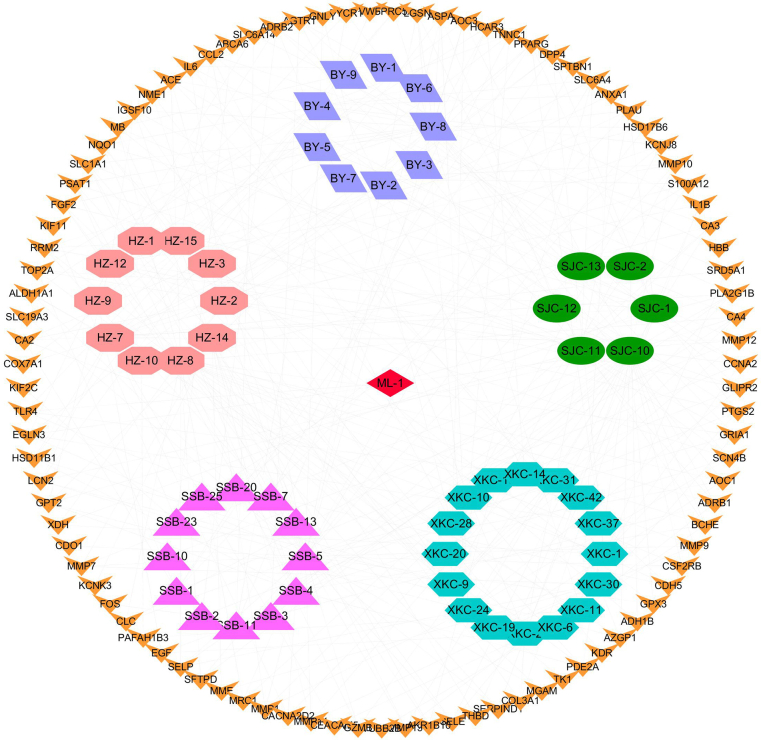


## Abbreviations

JLSJ: Juan-Liu-San-Jie
PPI: Protein-Protein Interaction
NSCLC: Non-small cell lung cancer
TCM: Traditional Chinese Medicine
MDSC: Myeloid-derived Suppressor Cell
UPLC: Ultra Performance Liquid Chromatography
DEG: Differentially Expressed Gene
BP: Biological Process
CC: Cellular Component
MF: Molecular Function
PVDF: Polyvinylidene Fluoride
XIAP: X-linked Inhibitor of Apoptosis Protein
