# Supplementary material for: Elucidation of the anti-lung cancer mechanism of Juan-Liu-San-Jie prescription based on network pharmacology and experimental validation
Source: Heliyon. 2023 Jul 23;9(8):e18298. doi: 10.1016/j.heliyon.2023.e18298 (PMC10407049; doi:10.1016/j.heliyon.2023.e18298)
Supplement: Multimedia component 2 [file mmc2.docx]

**Supplementary Table 1**

Information of 50 compounds identified from JLSJ prescription by UPLC/QTOF MS.

| **No.** | **Identification status** | **Observed m/z** | **Mass error (mDa)** | **Observed RT (min)** | **Theoretical Fragments Found** | **Adducts** | **TCM** |
| --- | --- | --- | --- | --- | --- | --- | --- |
| 1 | danshensu isomer | 221.0413 | -0.8 | 0.93 | 0 | +Na | HZ |
| 2 | pentose | 195.0511 | 0.1 | 0.95 | 1 | +HCOO, -H2O+HCOO | SJC/SSB/BY/XKC |
| 3 | valine | 118.0857 | -0.5 | 0.96 | 1 | +H | SJC/SSB/BY/XKC/HZ |
| 4 | glucose | 203.0517 | -0.9 | 0.99 | 0 | +Na | ML/SSB/BY |
| 5 | proline | 138.0544 | 1.8 | 1.08 | 1 | +Na, +H | BY |
| 6 | shikimic acid | 173.0457 | 0.2 | 1.56 | 4 | -H, -H2O-H | SSB |
| 7 | glutamate | 130.0492 | -0.6 | 2.76 | 1 | -H20+H | SJC/SSB/BY |
| 8 | phenylpropionic acid | 166.0856 | -0.7 | 4.8 | 1 | +H | SJC/SSB/BY/XKC |
| 9 | hordenine-O-α-L-rhamnoside | 312.1796 | -1 | 4.98 | 10 | +H | SSB |
| 10 | vitamin B1 | 301.0884 | 0 | 5.18 | 0 | +H | HZ |
| 11 | salvianolic acid D | 417.0805 | -2.2 | 5.4 | 7 | -H | BY |
| 12 | *cis*-caffeic acid | 163.0381 | -0.9 | 6.26 | 3 | -H20+H | BY |
| 13 | tryptophan | 205.0963 | -0.9 | 6.28 | 10 | +H | BY/XKC |
| 14 | 6-methoxy-7-hydroxycoumarin | 175.0382 | -0.8 | 6.87 | 1 | -H20+H, +H | BY |
| 15 | macrophyllin | 137.0242 | -0.2 | 6.89 | 2 | -H2O-H | SJC/BY |
| 16 | miltiron | 265.1541 | -4.5 | 7.03 | 0 | -H20+H | BY |
| 17 | *cis*-caffeic acid isomer | 163.0382 | -0.8 | 7.34 | 3 | -H20+H | BY |
| 18 | *cis*-caffeic acid isomer | 239.0557 | -0.4 | 7.54 | 7 | +HCOO | SSB |
| 19 | *cis*-caffeic acid isomer | 193.0506 | 0 | 7.8 | 3 | -H | SJC |
| 20 | danshensu isomer | 179.0351 | 0.1 | 7.98 | 4 | -H2O-H | SJC/BY |
| 21 | salvianolic acid D | 463.0912 | 3 | 9.06 | 22 | +HCOO, -H | XKC |
| 22 | 6-methoxy-7-hydroxycoumarin | 175.0382 | -0.8 | 9.07 | 3 | -H20+H, +H | BY |
| 23 | quercetin | 303.0493 | -0.7 | 9.86 | 2 | +H | SJC/XKC |
| 24 | quercetin-3-O-β-D-glucopyranoside | 465.1017 | -1 | 10.08 | 2 | +H | XKC |
| 25 | 6-methoxy-7-hydroxycoumarin | 193.0491 | -0.4 | 10.14 | 2 | +H | BY |
| 26 | salvianolic acid D | 463.0876 | -0.6 | 10.19 | 12 | +HCOO | BY |
| 27 | *cis*-caffeic acid | 163.0385 | -0.4 | 10.33 | 3 | -H20+H, +H | XKC |
| 28 | isopolygonin | 447.093 | -0.3 | 10.97 | 6 | -H | SJC |
| 29 | rosmarinic acid | 359.0772 | 0 | 11.56 | 20 | -H | BY |
| 30 | salvianolic acid B isomer | 717.1451 | -1 | 11.58 | 18 | -H, +HCOO | BY |
| 31 | salvianolic acid A | 493.1136 | -0.4 | 11.67 | 19 | -H | SJC |
| 32 | salvianolic acid B isomer | 717.1449 | -1.2 | 12.21 | 26 | -H | SJC |
| 33 | arjunglucoside I | 711.394 | -2.1 | 12.44 | 11 | +HCOO, -H | SJC/BY |
| 34 | salvianolic acid B isomer | 717.1435 | -2.7 | 12.53 | 12 | -H | BY |
| 35 | violet acid | 519.0927 | -0.6 | 13.1 | 6 | -H2O-H | SJC |
| 36 | salvianolic acid B isomer | 717.1443 | -1.9 | 13.24 | 22 | -H | SJC |
| 37 | (-) - liriodendron resin alcohol A | 401.1579 | -1.5 | 13.73 | 6 | -H20+H, +H | SJC/SSB/BY |
| 38 | lycium barbarum C | 964.418 | 0.4 | 13.92 | 49 | +Na | SSB/BY |
| 39 | (+) - siraitia grosvenori | 357.1343 | -0.1 | 15.1 | 3 | -H | SSB |
| 40 | methyl hexadecanoate | 315.2532 | -0.8 | 22.8 | 1 | +HCOO | SJC |
| 41 | hexadecanoic acid | 279.2309 | 1.4 | 23.9 | 0 | +Na | SJC/SSB/BY/XKC/HZ |
| 42 | kayaflavone | 581.143 | -1.2 | 24.05 | 2 | +H | SSB |
| 43 | methyl hexadecanoate | 297.2439 | 0.4 | 24.5 | 1 | -H2O+HCOO | HZ/SJC |
| 44 | 7,4',7',4'-tetra-O-methyl amantoflavin | 595.1594 | -0.5 | 24.95 | 1 | +H | SSB |
| 45 | oleanolic acid isomer | 455.3522 | -0.9 | 25.9 | 1 | -H | SJC/BY |
| 46 | oleanolic acid isomer | 455.3523 | -0.7 | 26.12 | 1 | -H, +HCOO | SJC/BY |
| 47 | oleanolic acid isomer | 455.3524 | -0.7 | 26.19 | 0 | -H, +HCOO | SJC/BY |
| 48 | hexadecanoic acid | 255.2332 | 0.2 | 27.14 | 0 | -H | SJC/BY/XKC |
| 49 | tanshinone diphenol isomer | 297.1532 | 3.5 | 27.45 | 1 | -H | HZ/ML/BY |
| 50 | tanshinone diphenol isomer | 297.1532 | 3.6 | 27.86 | 0 | -H | SSB |

Note: **SSB**(Shi-Shang-Bai): *Selaginella doederleinii* Hieron. ; **SJC**(Shi-Jian-Chuan): *Salvia chinensis* Herba. ; **BY**(Bai-Ying): *Solanum lyratum* Thunb; **XKC**(Xia-Ku-Cao): *Prunella vulgaris* L.; **HZ**(Hai-Zao): *Sargassum Pallidum (Turn.) C.Ag.;* and **ML**(Mu-Li): *Ostrea riuularis Gould.*
